# Beyond Permanent Genome Editing: Molecular Delivery Strategies for RNA Editing and Epigenome-Editing Therapeutics

**DOI:** 10.3390/ijms27146467

**Published:** 2026-07-21

**Authors:** Wajid Zaman, Asma Ayaz

**Affiliations:** 1 Department of Life Sciences, Yeungnam University, Gyeongsan 38541, Republic of Korea; 2 Faculty of Sports Science, Ningbo University, Ningbo 315211, China; asma@nbu.edu.cn

**Keywords:** reversible genetic medicines, targeted delivery, RNA editing, epigenome editing, nucleic acid therapeutics, intracellular delivery

## Abstract

Reversible genetic medicines are emerging as controllable alternatives to permanent genome editing by enabling programmable modulation of RNA sequence, transcript abundance, chromatin state, and gene expression without irreversible genomic alteration. However, reversibility is not a single binary property: transient editor exposure, decay of the molecular effect, recovery of cellular function, and clinical capacity to stop, redose, or counteract treatment may diverge. This review therefore distinguishes mechanistic, functional, and clinical reversibility while examining targeted delivery systems for RNA-editing and epigenome-editing therapeutics. Key payloads include ADAR-recruiting oligonucleotides, CRISPR-Cas13 RNA editors, guide RNAs, chemically modified RNAs, editor-encoding mRNAs, dCas9 transcriptional regulators, DNA methylation editors, histone-modifying systems, and CRISPRoff-like platforms. We evaluate extracellular and intracellular delivery barriers, including nuclease degradation, immune recognition, renal clearance, liver uptake, cellular entry, endosomal escape, cytoplasmic release, nuclear localization, chromatin access, editing-window duration, off-target activity, immunogenicity, repeat-dosing feasibility, manufacturing, quality control, potency assays, and regulatory translation. Overall, delivery systems for reversible genetic medicines should be judged by tissue selectivity, functional editing, duration of action, reversibility after treatment withdrawal, safety, manufacturability, and clinical controllability.

## 1. Introduction

Genetic medicines have transformed the conceptual boundaries of drug delivery by enabling therapeutic intervention at the level of nucleic acids, gene expression, and molecular information flow rather than only at proteins or signaling pathways [1]. Classical gene therapy and genome-editing approaches offer the possibility of long-lasting correction of disease-causing defects, particularly in monogenic disorders, cancer, immune diseases, and inherited metabolic conditions [2]. However, permanent genome modification also introduces major translational and safety concerns, including off-target editing, delivery barriers, and difficulty controlling long-term outcomes [3]. Once a genomic edit is introduced, the biological effect may be difficult or impossible to reverse, especially when the edited cell population expands, differentiates, or persists long term [4]. This creates particular concern for tissues with regenerative capacity, developing organs, immune cells, germline-adjacent contexts, and diseases in which only temporary modulation is needed [3].

The limitations of permanent genome editing are not restricted to irreversibility alone. Off-target DNA modification, unintended insertions or deletions, chromosomal rearrangements, mosaic editing, variable editing efficiency, and heterogeneous cellular responses can complicate therapeutic control [5]. Immunogenicity against editing enzymes, viral vectors, delivery vehicles, or newly expressed proteins may further restrict repeat dosing and long-term safety [6]. Dose control is also challenging because editing outcomes depend not only on the administered amount but also on delivery efficiency, intracellular trafficking, nuclease expression duration, DNA repair pathway activity, cell-cycle state, and tissue accessibility [7]. These uncertainties are particularly important when the therapeutic target is not a fixed genetic defect but a dynamic disease process such as inflammation, cancer signaling, fibrosis, neurodegeneration, or immune dysregulation [8]. In such settings, permanent editing may exceed the required therapeutic intervention and introduce ethical or safety concerns disproportionate to the clinical need [3].

Reversible genetic medicines provide an alternative therapeutic logic by modulating RNA sequence, RNA stability, translation, chromatin state, or transcriptional activity without permanently altering genomic DNA sequence [9,10]. RNA-editing systems can correct or recode transcripts at the RNA level, allowing transient modification of disease-relevant messages while preserving the underlying genome [11,12]. ADAR-recruiting oligonucleotides, guide RNA-directed RNA editors, CRISPR-Cas13-based systems, chemically modified RNAs, and editor-encoding mRNA platforms represent important approaches within this space [13,14,15]. Epigenome-editing systems provide another layer of reversible or tunable regulation by altering gene expression through DNA methylation, histone modification, chromatin remodeling, transcriptional repression, or transcriptional activation without cutting genomic DNA [16,17]. Similarly, transient gene silencing, mRNA degradation, splice modulation, and transcriptional control platforms can reduce, restore, or reshape gene expression for a defined therapeutic window [12,18].

The delivery requirements of reversible genetic medicines differ fundamentally from those of conventional small molecules, biologics, and permanent genome editors [19]. These payloads are often large, charged, nuclease-sensitive, immunostimulatory, and dependent on precise intracellular localization [19,20]. RNA editors may need cytoplasmic or nuclear access depending on the target transcript and editing mechanism, whereas epigenome editors usually require nuclear delivery and sustained but controllable residence at chromatin targets [10,15]. Payload format strongly influences delivery burden: oligonucleotides, mRNA, self-amplifying RNA, protein, ribonucleoprotein complexes, plasmid DNA, viral vectors, lipid nanoparticles, polymeric carriers, peptide systems, and extracellular vesicle-inspired platforms each differ in stability, tissue tropism, endosomal escape, duration of expression, repeat-dosing feasibility, and safety profile [20,21,22]. Therefore, reversible genetic-medicine delivery must be designed around payload architecture, route of administration, intracellular trafficking, editing kinetics, and reversibility rather than transfection efficiency alone [19].

A central challenge is controlling not only where an editor is delivered, but also how long the editor and its molecular consequences remain active [10,11,14]. For this review, reversibility is treated at three distinct but related levels: mechanistic reversibility, defined by clearance, degradation, dilution, inactivation, or externally controlled shutdown of the payload and delivery components; functional reversibility, defined by return of edited RNA, chromatin marks, gene-expression output, or phenotype toward a prespecified baseline after treatment withdrawal; and clinical reversibility, defined by the practical capacity to titrate, interrupt, redose, or counteract treatment without unacceptable delayed toxicity [23,24]. These levels may diverge. A transient mRNA or ribonucleoprotein exposure may generate effects that persist through transcript, protein, or chromatin turnover, whereas a delivery system may be redosable even when the induced molecular state is slow to reverse. Accordingly, delivery platforms should be evaluated as temporal control systems, not solely as transport vehicles.

This review critically examines delivery systems for reversible genetic medicines, with emphasis on RNA-editing and epigenome-editing therapeutics. It defines the major payload classes and evaluates extracellular, cellular, cytoplasmic, nuclear, and chromatin barriers to functional delivery. It then compares lipid nanoparticles, polymeric and peptide carriers, hybrid nanoplatforms, viral vectors, and extracellular vesicle-inspired systems in relation to tissue tropism, intracellular access, editing-window control, safety, repeat dosing, and manufacturability. The organizing perspective is that platform selection should integrate tissue selectivity, functional intracellular delivery, editing kinetics, mechanistic, functional, and clinical reversibility, safety, manufacturing control, and clinical manageability rather than transfection or transduction efficiency alone [19,24,25].

## 2. Literature Search Approach

This narrative review was informed by targeted searches of PubMed, Web of Science Core Collection, Scopus, and Google Scholar covering 1 January 2010 through 30 June 2026; earlier foundational studies were retained when necessary to establish mechanisms or platform history. Search concepts were combined iteratively using the following principal blocks: (“RNA editing” OR ADAR OR “ADAR-recruiting” OR LEAPER OR RESTORE OR Cas13 OR “RNA base editor”) AND (delivery OR nanoparticle OR “lipid nanoparticle” OR polymer* OR peptide OR viral OR “extracellular vesicle”); (“epigenome editing” OR “epigenetic editing” OR dCas9 OR CRISPRoff OR CRISPRon OR “DNA methylation editor” OR “histone editor” OR CRISPRa OR CRISPRi) AND (delivery OR “in vivo” OR nanoparticle OR mRNA OR RNP OR AAV); and (reversib* OR transient OR inducible OR “off switch” OR redosing OR “editing window”) AND (“genetic medicine” OR “RNA editing” OR “epigenome editing”). English-language, peer-reviewed primary studies and reviews directly addressing delivery, temporal control, safety, manufacturing, or regulatory translation were prioritized. Preprints were considered only when no peer-reviewed alternative was available and were identified as such; permanent DNA-editing studies were included only when they supplied a directly relevant delivery or controllability benchmark. Titles and abstracts were assessed first, followed by full-text evaluation when eligibility or quantitative interpretation required clarification. Because the search was iterative and not preregistered, query-level record counts were not prospectively logged and were not retrospectively reconstructed.

## 3. Therapeutic Payloads for Reversible Gene Modulation

Therapeutic payloads for reversible gene modulation differ substantially in molecular size, stability, intracellular site of action, duration of activity, immunogenicity, and delivery complexity [26]. Unlike permanent genome-editing systems, these payloads are designed to modify RNA sequence, transcript abundance, chromatin state, or gene-expression output without producing irreversible genomic DNA changes [23,27]. This reversibility is therapeutically attractive, but it places strong demands on delivery design because the payload must reach the correct tissue, enter the appropriate intracellular compartment, remain active for a controlled period, and then decline before excessive or off-target modulation occurs [11,28]. RNA-editing systems require efficient transcript access and protection from nuclease degradation, whereas epigenome-editing systems require nuclear delivery and controlled residence at regulatory DNA or chromatin regions [24,29]. Payload format further determines delivery burden: oligonucleotides, mRNA, self-amplifying RNA, protein, ribonucleoprotein complexes, plasmid DNA, viral vectors, lipid nanoparticles, polymeric carriers, and extracellular vesicle-inspired systems each provide different balances between potency, duration, repeat dosing, manufacturability, and safety [30,31]. The main reversible genetic-medicine payloads and their delivery requirements are summarized in Table 1.

### 3.1. RNA-Editing Systems

RNA-editing systems are attractive because they allow sequence-level correction or recoding at the transcript level without changing genomic DNA [27]. ADAR-recruiting oligonucleotides represent one of the most important approaches [13]. These systems use engineered guide oligonucleotides to recruit endogenous adenosine deaminases acting on RNA, enabling site-directed adenosine-to-inosine conversion in target transcripts [9]. Their delivery requirements are shaped by oligonucleotide stability, chemical modification, tissue uptake, endosomal escape, intracellular localization, and target-transcript accessibility [29]. Chemically modified RNAs can improve nuclease resistance, binding affinity, pharmacokinetic behavior, and reduced immune activation, but excessive modification may alter editing efficiency or specificity [11]. This class of payload is comparatively compact and may be more amenable to repeat dosing than large editor-encoding systems, but it requires highly efficient cellular delivery and careful control of off-target transcript editing [32].

CRISPR-Cas13-based RNA editors and direct editor-delivery systems offer broader programmable RNA targeting but introduce a larger delivery burden [33]. Cas13 systems can be engineered for transcript knockdown, RNA editing, or modulation of RNA fate depending on the editor architecture and guide RNA design [34]. These platforms usually require delivery of guide RNAs together with editor protein, editor mRNA, or vector-encoded machinery [35]. Direct editor delivery as protein or ribonucleoprotein can reduce prolonged expression and improve temporal control, whereas mRNA-based delivery allows transient intracellular production of the editor [24]. However, these systems face challenges related to payload size, immune recognition, cytosolic access, guide stability, target specificity, and control of editing duration [36]. Therefore, RNA-editing delivery should be evaluated not only by editing percentage, but also by transcriptome-wide specificity, editing persistence, reversibility after payload clearance, and functional rescue of disease-relevant RNA defects [25].

### 3.2. Epigenome-Editing Systems

Epigenome-editing systems regulate gene expression without cutting DNA or permanently changing nucleotide sequence [23]. Many platforms are based on catalytically inactive Cas proteins, especially dCas9, fused to transcriptional repressors, activators, DNA methylation domains, demethylation-related domains, histone acetyltransferases, histone deacetylases, or other chromatin-modifying effectors [37]. These systems can silence, activate, or fine-tune gene expression by targeting promoters, enhancers, silencers, or other regulatory elements [38]. Their therapeutic relevance is strong for diseases in which abnormal gene expression, rather than irreversible mutation correction, is the desired intervention [39]. Examples include transient repression of pathogenic genes, reactivation of protective genes, modulation of inflammatory pathways, immune-cell reprogramming, fibrosis-associated gene control, and regulation of cancer-related transcriptional networks [40]. However, epigenome editing usually requires nuclear delivery, guide RNA expression or delivery, sufficient chromatin residence, and controlled duration of activity [24].

CRISPRoff-like systems and related transcriptional memory platforms illustrate both the promise and complexity of epigenome editing [23]. These systems can establish more durable gene repression through targeted epigenetic marks, but the therapeutic advantage of persistence must be balanced against reversibility and safety [10]. A fully reversible genetic medicine should allow expression control to be adjusted, stopped, or reversed if disease status changes or adverse effects appear [41]. This requires delivery systems that regulate editor dose, exposure time, tissue distribution, and intracellular persistence [24]. Epigenome editors also raise specific validation challenges because off-target effects may occur at regulatory regions without obvious DNA sequence alteration [28]. Therefore, assessment should include transcriptome profiling, epigenome-wide analysis, chromatin-target specificity, persistence of epigenetic marks, reversibility of expression changes, and functional recovery after editor clearance [23]. Delivery platforms for epigenome editing must therefore be judged by nuclear access, potency, temporal control, and epigenetic safety rather than gene-expression change alone [24].

### 3.3. Payload Format and Delivery Burden

Payload format is one of the most important determinants of delivery burden [26]. Oligonucleotide-based systems are relatively small and chemically tunable, but they are vulnerable to nuclease degradation, renal clearance, poor cellular uptake, and endosomal trapping unless chemically stabilized or formulated with appropriate delivery systems [29]. mRNA delivery enables transient intracellular production of RNA editors, epigenome editors, or regulatory proteins, and it is compatible with lipid nanoparticles and other non-viral carriers [42]. However, mRNA requires protection from degradation, efficient endosomal escape, control of innate immune activation, and optimization of translation duration [43]. Self-amplifying RNA can extend expression from a lower dose, but its larger size and stronger innate immune stimulation may complicate tolerability and formulation [30,43]. Protein and ribonucleoprotein delivery can provide shorter exposure and better temporal control, but these payloads are large, fragile, and difficult to deliver efficiently into the cytosol or nucleus [24].

Plasmid DNA and viral vectors can support stronger or longer expression, but they may reduce reversibility if expression persists beyond the intended therapeutic window [9]. Viral vectors offer high delivery efficiency and tissue tropism, but they are constrained by packaging capacity, immunogenicity, manufacturing complexity, repeat-dosing limitations, and long-term monitoring requirements [34]. Non-viral nanoparticles, including lipid nanoparticles, polymeric carriers, peptide-based systems, and hybrid platforms, are more flexible and potentially repeatable, but their tissue specificity and intracellular delivery efficiency remain major challenges [31,42]. For reversible genetic medicines, the ideal format depends on whether the clinical objective requires a brief editing pulse, repeated transient modulation, sustained but controllable repression, or local tissue-specific regulation [25,39]. As outlined in Table 1, each payload class imposes distinct requirements for stability, intracellular trafficking, duration control, safety testing, and manufacturability.

### 3.4. Operationalizing Mechanistic, Functional, and Clinical Reversibility

The three levels introduced in the Introduction provide an operational framework for interpreting delivery performance. Mechanistic reversibility asks whether the editor, guide, vector, carrier, or regulatory component is degraded, cleared, diluted, switched off, or otherwise rendered inactive after treatment. Functional reversibility asks whether the edited RNA population, transcript abundance, chromatin mark, gene-expression output, protein level, and relevant phenotype return toward a prespecified baseline after editor activity declines. Clinical reversibility asks whether treatment can be titrated, interrupted, redosed, rescued, or counteracted without unacceptable delayed toxicity. These dimensions are non-binary and need not coincide. An mRNA-delivered RNA editor may be mechanistically transient while edited transcripts or downstream proteins persist; conversely, transiently delivered CRISPRoff-like systems may establish chromatin states that persist through cell division [23,24]. Non-DNA-cutting interventions should therefore not be described as inherently reversible solely because the payload is transient.

Evidence of reversibility should be reported on a time axis and should include, where technically feasible, payload clearance or inactivation, decline of on-target editing or gene modulation after treatment withdrawal, absence of progressive off-target molecular effects, recovery or controlled stabilization of the relevant phenotype within a prespecified window, and demonstration of rescue or counter-editing when such a strategy is claimed. For RNA editors, transcript and protein turnover should be distinguished from editor clearance. For epigenome editors, persistence of DNA methylation, histone states, chromatin accessibility, and transcriptional memory should be measured separately. This framework is applied below to delivery platforms, disease contexts, and manufacturing and regulatory endpoints.

## 4. Delivery Barriers for RNA and Epigenome Editors

**Figure 1:**
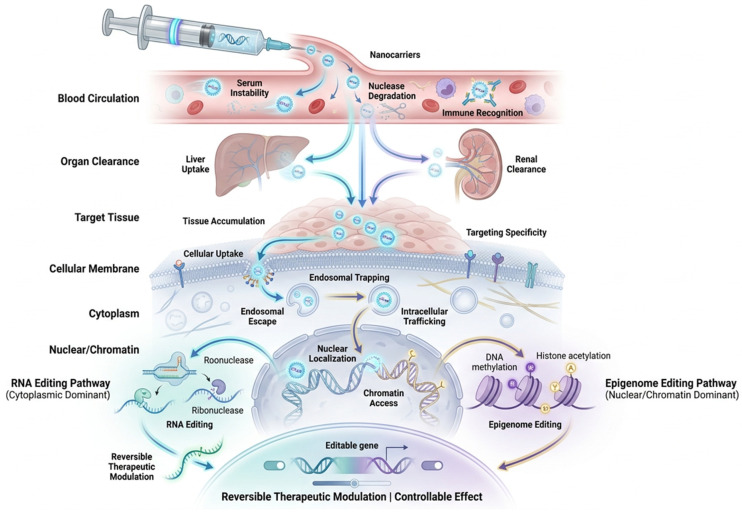
**Multilevel delivery barriers for reversible genetic medicines.** Schematic representation of the sequential barriers encountered by RNA-editing and epigenome-editing payloads, from systemic administration and blood circulation to serum instability, nuclease degradation, immune recognition, hepatic and renal clearance, target-tissue accumulation, cellular uptake, endosomal escape, intracellular trafficking, nuclear localization, chromatin access, and functional editing. The figure distinguishes the cytoplasmic RNA-editing pathway from the nuclear/chromatin-dependent epigenome-editing pathway, both leading to reversible therapeutic modulation. Created and edited using https://www.figurelabs.ai, accessed on 22 May 2026.

Delivery barriers for RNA and epigenome editors are more demanding than those for many conventional drug-delivery payloads because these systems must remain intact outside the cell, enter the correct tissue, escape intracellular compartments, and reach the appropriate molecular site of action [44]. Reversible genetic medicines are typically large, charged, structurally sensitive, and biologically active at very low intracellular concentrations [45]. Their therapeutic effect depends not only on tissue accumulation but also on functional intracellular availability [46]. A carrier may show favorable biodistribution yet fail therapeutically if the payload is degraded in serum, cleared by the kidney, sequestered in the liver, trapped in endosomes, excluded from the nucleus, or expressed for an inappropriate duration [46,47]. Therefore, delivery evaluation must distinguish between physical delivery to a tissue and functional delivery to the transcript, cytoplasm, nucleus, or chromatin target [48]. The integrated extracellular, systemic, cellular, and intracellular barrier landscape for RNA-editing and epigenome-editing delivery is illustrated in Figure 1.

### 4.1. Extracellular and Systemic Barriers

The first barrier is stability in extracellular and systemic environments [49]. RNA-editing guides, chemically modified RNAs, editor mRNA, self-amplifying RNA, ribonucleoproteins, and protein-based editors are vulnerable to degradation by nucleases, proteases, serum proteins, and inflammatory enzymes [50]. Naked RNA and oligonucleotides can be rapidly degraded or cleared before reaching target tissues unless chemically modified, conjugated, or encapsulated [47]. Serum proteins may bind delivery carriers and alter their circulation, biodistribution, immune recognition, and cellular uptake [48]. Larger editor systems may also activate innate immune pathways, particularly when RNA motifs, delivery lipids, viral vectors, or foreign protein components are recognized as danger-associated molecular structures [43]. Thus, extracellular stability must be considered together with immunological visibility and pharmacokinetic exposure [49].

Systemic biodistribution presents an additional barrier [51]. Many non-viral nanocarriers, especially lipid nanoparticles, show strong liver accumulation after intravenous administration, which is useful for hepatic targets but limiting for muscle, lung, heart, brain, kidney, immune-cell, or tumor applications [52,53]. Oligonucleotide-based payloads may also undergo renal clearance if they are small or insufficiently associated with carriers [47]. Viral vectors can provide tissue tropism, but their distribution is constrained by receptor expression, pre-existing immunity, capsid properties, dose, and route of administration [34]. For reversible genetic medicines, nonspecific biodistribution is especially concerning because off-target tissues may experience unwanted RNA editing, gene repression, gene activation, or immune stimulation [46]. Therefore, delivery systems must be evaluated for serum stability, nuclease resistance, immune recognition, renal clearance, liver uptake, target-tissue accumulation, and off-target biodistribution before editing efficiency is interpreted as therapeutically meaningful [25].

### 4.2. Cellular Entry and Endosomal Escape

Cellular entry is a major bottleneck because nucleic acids and editor complexes do not readily cross the plasma membrane [45]. Many delivery systems rely on receptor-mediated uptake, electrostatic interaction, lipid fusion, endocytosis, or membrane-disruptive mechanisms to enter cells [54]. Receptor-mediated uptake can improve cell specificity when the receptor is enriched in the target tissue, but receptor expression may vary by disease stage, cell type, and patient population [55]. Non-specific uptake can increase intracellular delivery but may also promote toxicity or off-target editing [28]. For RNA and epigenome editors, cellular uptake should not be interpreted as successful delivery unless the payload reaches the compartment where it can act [56]. A payload trapped at the membrane, within endosomes, or in lysosomes may contribute little to therapeutic editing even if total cellular fluorescence or uptake appears high.

Endosomal escape is one of the most important limitations in non-viral genetic-medicine delivery [56]. Lipid nanoparticles, polymeric carriers, peptide systems, dendrimers, and hybrid platforms may enter cells efficiently but release only a small fraction of their payload into the cytosol [57]. Endosomal trapping can lead to lysosomal degradation, loss of guide RNA activity, reduced editor expression, and poor functional bioavailability [58]. Strategies to improve escape include ionizable lipids, proton-sponge polymers, fusogenic peptides, pH-responsive materials, membrane-disruptive components, endosomolytic agents, and stimuli-responsive carriers [57,59]. However, increasing endosomal escape can also increase cytotoxicity, inflammation, and membrane damage [43]. Therefore, an optimal system balances escape efficiency with cellular safety [54]. Functional assays should measure editing, transcript correction, gene repression, protein restoration, or phenotypic rescue, not only uptake or fluorescence intensity [46].

### 4.3. Nuclear Versus Cytoplasmic Delivery Requirements

The intracellular destination differs between RNA-editing and epigenome-editing systems [24,27]. Many RNA editors act on transcripts in the cytoplasm, where mature mRNAs are translated and where transcript-level correction can directly influence protein output [60]. For such systems, cytosolic delivery and protection from cytoplasmic nucleases are central [32]. However, some RNA targets are nuclear, including pre-mRNAs, retained transcripts, splice-regulatory targets, and transcripts undergoing processing [61]. In these cases, RNA-editing systems may require nuclear access or delivery of components that can traffic between cytoplasm and nucleus [62]. The localization of the editor protein, guide RNA, and target transcript therefore determines whether cytoplasmic delivery is sufficient or whether nuclear trafficking becomes necessary [63].

Epigenome editors have stricter nuclear delivery requirements because they must access chromatin and regulatory DNA regions [64]. dCas9-based repressors, activators, DNA methylation editors, histone-modifying editors, and CRISPRoff-like systems require nuclear localization, guide RNA availability, chromatin binding, and sufficient residence time at promoters, enhancers, or other regulatory elements [17,23]. Nuclear entry is challenging for large proteins, mRNA-derived editor products, plasmid DNA, and ribonucleoprotein complexes [32]. It may require nuclear localization signals, vector-mediated expression, nuclear transport-compatible payload design, or delivery during cell-cycle states when nuclear access is more permissive [63]. At the same time, prolonged nuclear residence may increase off-target epigenetic effects or reduce reversibility [28]. Thus, intracellular delivery for reversible genetic medicines should be designed according to the required site of action: cytosolic transcript editing, nuclear transcript editing, chromatin-level repression, chromatin-level activation, or transient epigenetic memory [24].

## 5. Delivery Platforms for Transient Genetic Modulation

**Figure 2:**
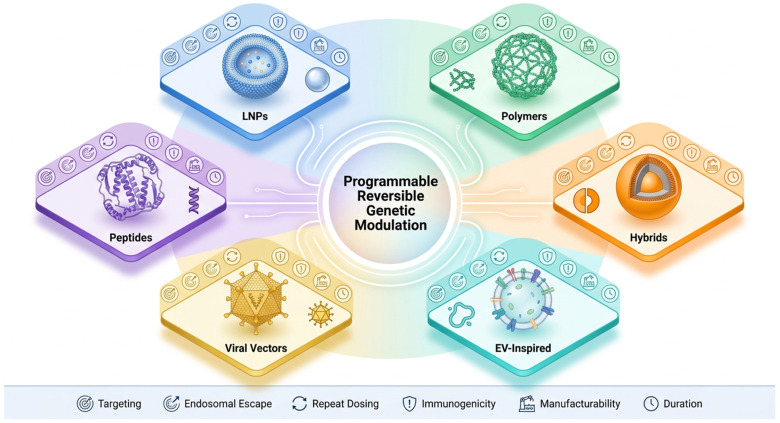
**Delivery-platform landscape for transient genetic modulation.** Overview of major delivery platforms for reversible genetic medicines, including lipid nanoparticles, polymers, peptide carriers, hybrid nanocarriers, viral vectors, and extracellular vesicle-inspired systems. The platform map highlights key design considerations such as targeting, endosomal escape, repeat-dosing feasibility, immunogenicity, manufacturability, and duration of activity. Created and edited using https://www.figurelabs.ai, accessed on 22 May 2026.

Delivery platforms for transient genetic modulation must be selected according to payload architecture, target tissue, intracellular destination, desired duration of activity, repeat-dosing feasibility, and safety requirements [65]. Unlike permanent genome-editing systems, reversible genetic medicines depend strongly on controlling the magnitude and duration of editor exposure [6]. A platform that produces prolonged expression may increase potency but reduce reversibility, whereas a rapidly cleared platform may improve safety but provide insufficient editing [66]. Therefore, the delivery vehicle is not only a transport system; it is also a regulator of editing kinetics, tissue selectivity, intracellular bioavailability, and therapeutic reversibility [67]. Lipid nanoparticles, polymeric carriers, peptide-based systems, hybrid nanocarriers, viral vectors, and extracellular vesicle-inspired platforms each offer distinct advantages and limitations for RNA-editing and epigenome-editing therapeutics [68]. The major delivery-platform classes for transient and reversible genetic modulation are compared in Figure 2.

### 5.1. Lipid Nanoparticles and Lipid-Based Carriers

Lipid nanoparticles are among the most advanced non-viral platforms for nucleic-acid delivery and are highly relevant for reversible genetic medicines [26]. Their success is largely based on the use of ionizable lipids, which remain relatively neutral at physiological pH but become protonated in acidic endosomal compartments, promoting endosomal destabilization and cytosolic release [69]. This property is particularly important for mRNA, guide RNA, RNA-editor systems, and oligonucleotide-based payloads that require protection from nucleases and functional intracellular delivery [45]. Helper lipids support particle structure and membrane fusion, cholesterol improves stability and lipid packing, and PEG-lipids regulate particle size, colloidal stability, circulation behavior, and aggregation [70]. Together, these components determine encapsulation efficiency, tissue distribution, immune activation, endosomal escape, and duration of expression [48].

For RNA-editor delivery, lipid nanoparticles can be used to deliver guide RNAs, editor-encoding mRNA, chemically modified RNA, or combinations of RNA payloads [25]. Their transient expression profile is attractive because it supports temporary editing and reduces the risk of prolonged off-target activity [34]. However, many lipid nanoparticles show preferential liver accumulation after systemic administration, which is useful for hepatic diseases but limiting for extrahepatic targets such as muscle, lung, brain, heart, kidney, immune cells, or solid tumors [53]. Tissue tropism can be modified through lipid composition, particle size, surface chemistry, route of administration, targeting ligands, and organ-selective lipid formulations, but extrahepatic delivery remains a major challenge [51,55]. Lipid-based carriers may also induce innate immune responses, complement activation, injection-site reactions, or inflammatory signaling depending on lipid structure, dose, route, and repeat administration [43]. Thus, LNPs are powerful platforms for transient genetic modulation, but their design must balance RNA protection, endosomal escape, tissue specificity, expression duration, and immunological tolerability [58].

Extrahepatic targeting must therefore be interpreted as a payload-dependent problem rather than a generic nanoparticle property. Compact oligonucleotide guides may be compatible with conjugation, local administration, or repeated systemic dosing when chemistry and immune activation are controlled [47,55]. In contrast, editor mRNA, self-amplifying RNA, ribonucleoprotein complexes, and dCas9-based epigenome editors require larger cargo capacity, efficient endosomal escape, and, for chromatin-directed systems, nuclear access [24,42]. Strategies such as route optimization, local injection, inhaled delivery, intrathecal delivery, ligand-mediated uptake, organ-selective lipid formulations, polymer–lipid hybrids, and engineered vesicle or viral tropism can broaden tissue reach, but each adds new constraints related to formulation reproducibility, immune recognition, and cell-type specificity [51,53]. Thus, broad disease applicability should be interpreted cautiously until functional delivery is demonstrated in the relevant target tissue and cell type.

### 5.2. Polymeric, Peptide, and Hybrid Nanocarriers

Polymeric nanocarriers provide flexible chemistry for delivering oligonucleotides, RNA, proteins, ribonucleoproteins, and plasmid-based systems [71]. Polyplexes formed through electrostatic interaction between cationic polymers and nucleic acids can protect payloads from degradation and promote cellular uptake [72]. Dendrimers offer highly branched architectures with tunable surface groups, multivalent binding, and potential for ligand attachment [73]. Biodegradable polymers can reduce long-term toxicity and allow controlled release, while stimuli-responsive polymers can trigger payload release in response to pH, redox conditions, enzymes, or other intracellular cues [31]. However, polymeric carriers often face challenges related to cytotoxicity, aggregation, serum instability, endosomal escape, and reproducibility [74]. For reversible genetic medicines, polymer degradation and payload release should be carefully controlled so that editing activity remains transient and does not persist beyond the intended therapeutic window [75].

Peptide-based delivery systems, including cell-penetrating peptides, fusogenic peptides, endosomolytic peptides, and targeting peptides, can improve cellular entry and intracellular trafficking [76]. They are especially useful when the payload is a protein editor, ribonucleoprotein complex, or oligonucleotide requiring cytosolic or nuclear access [62]. Cell-penetrating peptides can enhance membrane interaction, while fusogenic or pH-sensitive peptides can support endosomal escape [77]. However, peptide systems may suffer from proteolytic degradation, limited serum stability, immunogenicity, and nonspecific uptake [78]. Hybrid nanocarriers, such as polymer–lipid nanoparticles, lipid–peptide systems, polymer–peptide complexes, and inorganic–organic platforms, attempt to combine the strengths of multiple materials [68]. For example, a lipid component may improve membrane fusion, a polymeric core may stabilize the payload, and a peptide or ligand may enhance targeting or endosomal escape [79]. These hybrid systems are promising for reversible gene modulation because they allow modular tuning of stability, uptake, release, and intracellular trafficking, but they also increase manufacturing and quality-control complexity [80].

### 5.3. Viral and Extracellular Vesicle-Inspired Systems

Viral vectors remain important for genetic-medicine delivery because they provide high transduction efficiency and, in some cases, strong tissue tropism [81]. Adeno-associated virus is widely used because of its relatively favorable safety profile and ability to deliver genetic cargo to several tissues, including liver, muscle, retina, and nervous system [78]. However, its limited packaging capacity restricts delivery of large epigenome editors, multi-domain dCas9 systems, or complex RNA-editing machinery [34]. Pre-existing immunity, capsid immune responses, long-term expression, and repeat-dosing limitations also complicate its use for reversible therapies [82]. Lentiviral vectors can provide efficient gene delivery and stable expression, but integration into the host genome is generally less suitable for transient modulation when reversibility is required [83]. Adenoviral vectors can support larger cargo and strong expression, but immunogenicity and inflammatory responses remain important concerns [84]. Thus, viral vectors may be useful when durable expression is required, but they must be used cautiously when the therapeutic goal is temporary and reversible genetic modulation [85].

Extracellular vesicle-inspired systems and engineered exosome-like carriers offer an alternative biologically derived approach [86]. These platforms may provide natural membrane composition, improved biocompatibility, intercellular communication properties, and potential tissue-targeting features [87]. Engineered extracellular vesicles can carry RNAs, proteins, guide molecules, or regulatory payloads and may be modified to improve targeting, loading, or intracellular delivery [88]. They are attractive for reversible genetic medicines because they can potentially deliver fragile payloads with lower synthetic-material toxicity [89]. However, their translation is limited by challenges in scalable production, purification, cargo loading, batch reproducibility, potency testing, storage stability, and safety characterization [90]. In addition, endogenous vesicle components may produce biological effects independent of the intended genetic payload [91]. Therefore, viral and extracellular vesicle-inspired systems should be evaluated through the lens of reversibility, expression duration, immunogenicity, manufacturing feasibility, and regulatory controllability rather than delivery efficiency alone [92]. Table 2 summarizes the relationship between major payload–carrier combinations, dominant functional delivery barriers, intracellular destinations, tissue-tropism considerations, and redosing constraints. It highlights that physical biodistribution, cellular uptake, and functional editing represent distinct levels of delivery success and should not be treated as equivalent therapeutic endpoints.

Reversibility profile. AAV and integrating vectors may remain mechanistically persistent and are often difficult to redose because of pre-existing or treatment-induced immunity; consequently, durable expression can conflict with clinical reversibility even when the encoded effector does not cut DNA. Extracellular vesicle-inspired systems may support shorter exposure, but variable loading, endogenous cargo, and batch heterogeneity complicate prediction of functional washout. Route restriction, transient payload formats, and predefined rescue or counter-regulation plans are therefore particularly important for these platforms [82,83,90].

## 6. Controlling Duration, Dose, and Reversibility

**Figure 3:**
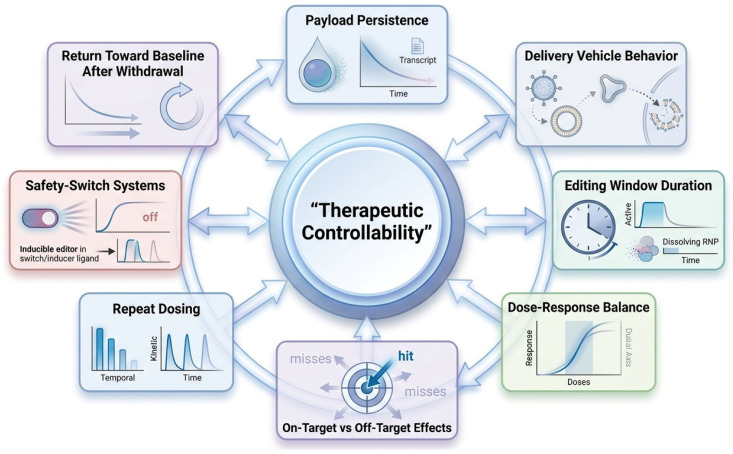
**Systems-control framework for editing duration, dose, and reversibility.** Conceptual framework showing how delivery systems regulate therapeutic controllability through payload persistence, delivery-vehicle behavior, editing-window duration, dose–response balance, repeat dosing, safety-switch systems, on-target versus off-target effects, and return toward baseline after treatment withdrawal. The figure emphasizes that delivery systems function as regulators of genetic-medicine activity, not only as transport vehicles. Created and edited using https://www.figurelabs.ai, accessed on 22 May 2026.

Controlling duration, dose, and reversibility is central to the therapeutic logic of reversible genetic medicines [93]. Unlike permanent genome-editing approaches, RNA-editing and epigenome-editing systems are intended to produce a tunable biological effect that persists only as long as clinically required [27,94]. This makes delivery design inseparable from temporal control [66]. A formulation should not only transport an editor to the correct tissue and intracellular compartment, but should also define how long the payload remains stable, how much editor activity is produced, how broadly the target is modified, and whether the effect declines after treatment withdrawal [95]. For reversible genetic modulation, excessive persistence can be as problematic as insufficient delivery [96]. Prolonged RNA editing may increase off-target transcript modification, whereas persistent epigenetic repression or activation may produce unintended gene-expression changes that outlast the intended therapeutic window [28,97]. Therefore, duration, dose, and reversibility should be treated as critical performance attributes of delivery systems rather than secondary pharmacological outcomes [24]. A systems-level framework linking delivery design to editing duration, dose response, safety, and reversibility is shown in Figure 3.

### 6.1. Transient Expression and Editing-Window Control

Transient expression is a major advantage of non-integrating genetic-medicine platforms, but it must be carefully controlled [98]. Editor activity depends on the stability and persistence of the delivered payload, including mRNA half-life, self-amplifying RNA duration, guide RNA stability, protein degradation rate, ribonucleoprotein persistence, vector expression kinetics, and intracellular degradation pathways [43,99]. mRNA-based delivery can support temporary production of RNA editors or epigenome editors, while chemical modifications, untranslated regions, cap structure, poly(A) tail length, and formulation environment can influence translation efficiency and expression duration [26]. Protein or ribonucleoprotein delivery can provide a shorter editing window because the active editor is delivered directly and degraded over time, reducing the risk of prolonged activity [66]. In contrast, plasmid DNA and viral vectors may produce longer expression, which can improve potency but may reduce reversibility if expression persists beyond therapeutic need [98].

Editing-window control is especially important because different diseases require different durations of gene modulation [25]. A transient correction of a pathogenic transcript may be sufficient for short-term metabolic rescue, inflammatory suppression, or temporary protein restoration [27]. In contrast, chronic diseases may require repeated dosing or longer but still controllable modulation [93]. For epigenome editing, duration control is more complex because the editor may be transient, while the induced chromatin state may persist [23]. This can be beneficial when durable repression or activation is desired, but it can also compromise reversibility [41]. Delivery systems should therefore be designed according to the required therapeutic window: brief pulse editing, repeated transient modulation, sustained but reversible expression control, or locally restricted genetic regulation [24]. The most appropriate platform depends on whether the therapeutic objective prioritizes rapid onset, short exposure, prolonged benefit, repeat dosing, or epigenetic memory [39].

### 6.2. Dose–Response and Therapeutic Window

Dose–response control is a major determinant of safety and efficacy in reversible genetic-medicine delivery [96]. Under-editing may fail to restore sufficient protein function, suppress a pathogenic transcript, or modify disease-relevant gene expression [25]. Over-editing may cause excessive pathway suppression, unintended gain or loss of function, cellular stress, toxicity, immune activation, or disruption of normal transcriptome and epigenome balance [28,36]. Unlike conventional drugs, the relationship between administered dose and biological effect may be nonlinear because editing depends on delivery efficiency, endosomal escape, editor expression, guide abundance, target accessibility, enzymatic activity, and turnover of edited RNA or epigenetic marks [46]. Therefore, the therapeutic window should be defined by functional correction and safety, not only by amount of payload administered [100].

Off-target activity is closely linked to dose and exposure duration [101]. Externally gated RNA-editing systems, including photoactivatable CRISPR-Cas13 base editing and small-molecule-inducible or photoactivatable RNA N1-methyladenosine editing, provide complementary strategies for constraining the editing window [102,103]. Higher doses, prolonged expression, persistent guide RNAs, or repeated administration may increase the probability of unintended RNA editing, transcript knockdown, chromatin modification, immune activation, or tissue toxicity [43,97]. Repeat dosing is particularly relevant for reversible therapies because temporary effects may require periodic re-administration [11]. However, repeated exposure can also increase immunogenicity, anti-vector responses, complement activation, inflammatory reactions, or reduced delivery efficiency [81]. Exposure control therefore requires careful optimization of dose level, dosing interval, payload format, carrier clearance, editor persistence, and reversibility of biological effect [96]. A robust delivery system should achieve sufficient on-target modulation while minimizing off-target editing, excessive duration, and cumulative toxicity [100].

Off-target activity is closely linked to dose and exposure duration [101]. Higher doses, prolonged expression, persistent guide RNAs, or repeated administration may increase the probability of unintended RNA editing, transcript knockdown, chromatin modification, immune activation, or tissue toxicity [43,97]. Repeat dosing should therefore be treated as a conditional advantage rather than a universal feature of reversible platforms. It is most realistic for chemically stabilized oligonucleotides, short RNAs, and some non-viral carriers when complement activation, anti-PEG or anti-carrier responses, cytokine induction, and tissue accumulation can be controlled [47,104]. It is more restricted for AAV and other viral vectors because pre-existing immunity and treatment-induced anti-capsid responses can reduce efficacy and complicate re-administration [82]. Editor proteins and ribonucleoproteins may provide short exposure, but repeated dosing requires evaluation of anti-editor antibodies, cellular immunity, and inflammatory priming [24]. Thus, redosing should be supported by exposure-response data, immune monitoring, and evidence that repeated treatment does not broaden off-target editing or epigenetic perturbation.

### 6.3. Safety Switches and Reversibility Strategies

Safety switches and reversibility strategies provide additional control over genetic-medicine activity [93]. Named examples include orthogonal small-molecule-inducible Cas13 circuits for programmable RNA regulation [93], the FIRE-Cas9 architecture for rapid and reversible recruitment of endogenous chromatin regulators [94], and chemical-inducible Cas9 systems that couple activity to externally controlled dimerization [96]. Related designs can regulate editor abundance, protein stability, nuclear localization, guide availability, or effector assembly. Self-limiting vectors, degradable carriers, short-lived mRNA, and directly delivered ribonucleoproteins can further restrict exposure [99,105]. These mechanisms should be validated by trigger-response kinetics, residual activity after trigger withdrawal, leakiness in the off state, and the capacity to restore the relevant molecular or phenotypic endpoint.

Reversible epigenetic modulation requires special attention because chromatin effects may persist after editor clearance [23]. CRISPRoff and CRISPRon demonstrate programmable deposition and reversal of transcriptional memory [23], whereas transient RNP delivery with RENDER shows that a short mechanistic exposure can still produce long-lasting silencing [24]. Depending on the clinical objective, persistence may reduce dosing frequency or may create risk when the target has context-dependent functions. Development programs should therefore specify a counter-editing or rescue strategy, measure locus-specific and epigenome-wide washout, and define a clinically acceptable time to functional recovery rather than inferring reversibility from non-integrating delivery alone [28,94].

## 7. Disease Applications of Reversible Genetic-Medicine Delivery

Reversible genetic-medicine delivery has broad therapeutic relevance because many diseases require temporary, tunable, or repeatable modulation of gene expression rather than permanent genome correction [27,93]. In some inherited disorders, transcript-level repair or splice modulation may restore sufficient protein function without altering genomic DNA [25,106]. In cancer, inflammation, immune disease, fibrosis, neurodegeneration, and aging-related disorders, disease mechanisms are often dynamic, context-dependent, and stage-specific, making reversible regulation more appropriate than irreversible editing [39,107]. The key advantage of RNA editing and epigenome editing is that therapeutic intervention can be adjusted according to disease activity, tissue response, safety signals, and treatment duration [41,94]. However, each disease area imposes distinct delivery requirements, including tissue tropism, cellular specificity, editing-window control, immune tolerability, repeat dosing, and access to cytoplasmic or nuclear targets [108,109]. Major disease applications, editable targets, and delivery strategies are summarized in Table 3.

### 7.1. Genetic and Rare Diseases

Genetic and rare diseases are strong candidates for reversible genetic-medicine delivery when the pathogenic mechanism can be corrected at the RNA or gene-expression level [25]. Nonsense mutations may be addressed through RNA editing that converts premature stop codons or restores coding potential at the transcript level, while splice defects may be corrected by guide RNAs, antisense oligonucleotides, splice-modulating systems, or RNA-targeted editors [25,110]. Such approaches are attractive because they avoid permanent alteration of the genome while allowing repeated or adjustable correction of the disease-relevant transcript [27]. Metabolic disorders and liver diseases are particularly relevant because the liver is comparatively accessible to lipid nanoparticles, oligonucleotides, and several viral or non-viral delivery platforms [111]. For hepatic enzyme deficiencies, transient transcript correction, gene activation, or repression of toxic gain-of-function transcripts may provide clinically meaningful benefit if sufficient hepatocyte delivery and repeat dosing are achieved [111].

Muscle diseases and inherited neurological disorders present greater delivery challenges because tissue access is more difficult and the therapeutic target may be widely distributed [106]. Skeletal muscle requires broad tissue coverage, while inherited neurological disorders require crossing or bypassing the blood–brain barrier and achieving cell-type-specific delivery to neurons, glia, or supporting cells [112,113]. RNA editing may be useful when correction of a mutant transcript is sufficient, whereas epigenome editing may help activate compensatory genes, repress toxic transcripts, or modulate disease pathways [24,27]. However, safety is especially important in rare genetic diseases because many patients require long-term treatment [106]. Delivery systems must therefore support durable but controllable benefit, low immunogenicity, repeat administration, and minimal off-target editing across the transcriptome or epigenome [11]. In these indications, mechanistic clearance should be paired with evidence that transcript and protein correction declines predictably, that redosing restores activity without cumulative toxicity, and that clinical rescue criteria are linked to disease recurrence rather than payload disappearance alone.

### 7.2. Cancer, Inflammation, and Immune Diseases

Cancer applications of reversible genetic medicines are conceptually distinct from inherited disease applications because the goal is often to modulate dynamic oncogenic signaling rather than permanently correct a single mutation [39]. Transient oncogene repression, restoration of tumor-suppressive pathways, modulation of drug-resistance genes, inhibition of metastatic programs, and regulation of tumor microenvironment signals may all be approached through RNA-targeted or epigenome-targeted systems [40,114]. Reversible modulation is attractive in cancer because many targets are context-dependent; excessive or permanent repression of genes with normal physiological roles may increase toxicity [39]. Delivery strategies may include lipid nanoparticles, polymeric carriers, tumor-targeted nanoparticles, local depots, engineered immune cells, or viral vectors, depending on whether the target is a tumor cell, stromal cell, immune cell, or local microenvironment [42,115]. For cancer therapy, delivery must be evaluated not only by editing efficiency but also by tumor penetration, cell-type specificity, immune activation, and reversibility after treatment discontinuation [39].

Inflammation and immune diseases also benefit from reversible genetic modulation because immune pathways are highly dynamic and require balanced control rather than permanent suppression [107]. RNA editing or epigenome editing could transiently reduce pathogenic cytokine expression, reprogram immune-cell activation states, modulate checkpoint molecules, or adjust inflammatory signaling pathways [116,117]. In autoimmune diseases, inflammatory bowel disease, arthritis, asthma, allergic disease, and transplant-related immune activation, temporary repression of disease-driving mediators may reduce pathology while preserving essential immune defense [107,118]. Immune-cell reprogramming is especially relevant for ex vivo or in vivo delivery to T cells, macrophages, dendritic cells, or regulatory immune populations [117]. However, immune-targeted delivery has strict safety requirements because unintended editing can impair host defense, promote immune imbalance, or trigger excessive immunosuppression [104]. Therefore, exposure control, cell specificity, cytokine monitoring, and reversibility are central to therapeutic development [119]. Because inflammatory and immune states can change rapidly, functional recovery after treatment withdrawal, cytokine and complement monitoring, and the absence of cumulative immune priming should be prespecified as clinical-reversibility endpoints.

### 7.3. Neurological, Cardiovascular, and Age-Related Diseases

Neurological diseases represent one of the most important but technically demanding areas for reversible genetic-medicine delivery [109]. Disorders involving toxic protein accumulation, altered RNA processing, neuroinflammation, synaptic dysfunction, or abnormal gene expression may benefit from transient modulation of disease-associated transcripts [120]. For example, reducing tau, alpha-synuclein, mutant huntingtin, or other pathogenic transcripts may be therapeutically useful if delivery reaches the relevant neuronal or glial populations and avoids excessive suppression of physiological functions [109,120]. Epigenome editing may also be used to regulate neuroprotective genes, inflammatory pathways, or disease-associated transcriptional programs [24]. However, central nervous system delivery requires overcoming the blood–brain barrier, achieving regional or cell-type specificity, avoiding neurotoxicity, and carefully controlling duration because neuronal gene-expression changes may have long-lasting functional consequences [109,113].

Cardiovascular, fibrotic, and age-related diseases provide additional opportunities for reversible genetic modulation [108]. Cardiac remodeling, hypertrophy, vascular dysfunction, and heart failure involve dynamic transcriptional and epigenetic programs that may require temporary correction rather than permanent editing [121,122]. Fibrosis-associated genes in the heart, lung, liver, kidney, and skin may be targeted through transient repression of profibrotic pathways or activation of antifibrotic programs [111,123]. Age-related diseases and senescence-associated pathways may also benefit from controlled modulation of inflammatory, metabolic, mitochondrial, or senescence-related genes [124]. However, these applications require careful balancing because pathways involved in aging, repair, fibrosis, and inflammation often have protective roles in some contexts and harmful roles in others [125]. Reversible genetic-medicine delivery is therefore especially valuable in these diseases because it allows therapeutic modulation to be adjusted, repeated, or withdrawn according to tissue response and safety [93]. In post-mitotic or slowly renewing tissues, prolonged molecular follow-up is required because transient payload exposure may yield durable functional changes; local administration and validated counter-regulation strategies may therefore be more important than simple systemic clearance.

## 8. Manufacturing, Quality Control, and Regulatory Translation

Manufacturing, quality control, and regulatory translation are central to the clinical development of reversible genetic-medicine delivery systems because these products combine nucleic-acid payloads, editing machinery, delivery vehicles, intracellular trafficking requirements, and functional biological activity [97]. Unlike conventional formulations, quality cannot be defined only by particle size, encapsulation efficiency, or chemical stability [126]. A reversible genetic-medicine product must preserve RNA or protein integrity, deliver the payload to the correct tissue and intracellular compartment, produce predictable on-target modulation, limit off-target activity, and maintain reversibility within a defined therapeutic window [97,127]. This creates a development challenge in which formulation quality, editor potency, guide specificity, immune safety, biodistribution, repeat dosing, and long-term monitoring must be evaluated together [128].

### 8.1. Critical Quality Attributes of Editor Delivery Systems

Critical quality attributes for editor delivery systems include both carrier-related and payload-specific parameters [126]. For lipid nanoparticles, polymeric carriers, peptide systems, hybrid nanocarriers, extracellular vesicle-inspired platforms, and viral vectors, key formulation attributes include particle size, polydispersity, surface charge, morphology, encapsulation efficiency, payload loading, colloidal stability, sterility, endotoxin level, residual solvents, storage stability, and batch reproducibility [97,127]. However, reversible genetic medicines require additional molecular-quality attributes [129]. RNA integrity, guide RNA purity, mRNA capping efficiency, poly(A) tail quality, chemical modification consistency, protein integrity, ribonucleoprotein stability, editor activity, vector genome quality, and absence of truncated or degraded payloads are essential because small changes in payload quality can strongly affect editing efficiency, immune activation, and safety [129,130].

Potency is one of the most important but difficult quality attributes [131] (Table 4). A formulation may contain the correct amount of RNA or editor protein but still show poor functional activity if the payload is degraded, improperly complexed, inefficiently released from the carrier, or unable to reach the cytoplasm or nucleus [132]. Therefore, potency assays should measure functional editing or gene modulation rather than payload content alone [131]. For RNA editing, this may include target-transcript editing efficiency, restoration of protein function, correction of disease-relevant RNA defects, and decline of editing after payload clearance [27]. For epigenome editing, potency should include transcriptional repression or activation, target chromatin-mark change, durability of the intended expression effect, and reversibility or controlled persistence after editor withdrawal [10,132]. Release criteria should therefore incorporate temporal control, not only particle identity, purity, and initial activity. Stability testing should also evaluate whether storage affects particle properties, RNA integrity, guide activity, editor function, endosomal escape capacity, and biological potency [132]. Thus, quality control must connect physical formulation attributes with molecular activity, editing-window behavior, reversibility, and safety-relevant off-target readouts. Release and stability specifications should be mapped explicitly to the three reversibility levels: payload identity, integrity, and clearance inform mechanistic reversibility; washout of editing, transcriptional output, and phenotype informs functional reversibility; and reproducible interruption, redosing, rescue, and delayed-toxicity monitoring inform clinical reversibility. Potency assays should therefore include time-resolved onset, peak effect, decay after withdrawal, and return-to-baseline or controlled-stabilization criteria whenever a reversible claim is made.

### 8.2. Functional Validation and Safety Testing

Functional validation should establish that the delivery system produces the intended on-target effect at the correct molecular site and within the desired duration [27]. For RNA-editing systems, this includes quantifying editing at the target transcript, determining dose–response behavior, assessing editing persistence, and confirming functional correction at the protein or cellular phenotype level [15]. For epigenome editors, validation should confirm target-site engagement, transcriptional repression or activation, chromatin-state modification, duration of expression change, and reversibility after editor clearance or treatment withdrawal [10,41]. Functional testing should be performed in disease-relevant cellular models, primary cells, organoids, animal models, or tissue-specific systems where possible, because editing efficiency and specificity can differ substantially between simplified cell lines and clinically relevant tissues [24].

Safety testing is especially important because reversible genetic medicines may produce unintended molecular changes even without permanent DNA editing [97]. Minimum evidence standards should be modality-specific. For ADAR-recruiting guides, testing should quantify on-target editing, bystander editing within the target transcript, and transcriptome-wide A-to-I changes under clinically relevant exposure [13,29]. For Cas13-based systems, assessment should include guide-dependent off-target binding, unintended knockdown, altered splicing or RNA stability, and collateral activity where applicable [33,36]. For editor mRNA, self-amplifying RNA, protein, or ribonucleoprotein delivery, safety testing should link dose and exposure duration to off-target editing, innate immune activation, and cellular stress [24,43]. For epigenome editors, validation should include target-site chromatin assays together with epigenome-wide profiling to detect off-target methylation, histone-mark changes, chromatin remodeling, or unintended gene-expression programs [28,132,133]. Immunogenicity should be evaluated separately for the editor protein, RNA payload, delivery carrier, viral capsid, lipid or polymeric components, and for the immune consequences of repeated dosing [104]. Biodistribution studies should determine where the carrier and payload accumulate, which cell types are edited, and whether off-target tissues show molecular or functional changes [128]. Toxicity evaluation should include innate immune activation, complement activation, cytokine release, tissue inflammation, organ injury, and delayed effects after repeated or prolonged exposure [104].

### 8.3. Scale-Up and Regulatory Challenges

Scale-up presents major challenges because reversible genetic-medicine delivery systems are highly sensitive to manufacturing conditions [97]. Lipid nanoparticle manufacturing requires tight control of lipid composition, RNA-to-lipid ratio, mixing speed, solvent exchange, buffer conditions, particle formation, sterile filtration, and storage [127]. Small process changes can alter particle size, encapsulation, tissue tropism, endosomal escape, potency, and immune response [130,133]. Polymeric and hybrid carriers face similar challenges related to polymer molecular weight, charge ratio, complexation efficiency, residual solvent, aggregation, and reproducibility [126]. Viral vectors introduce additional constraints, including packaging capacity, vector genome integrity, capsid quality, empty-to-full particle ratio, infectivity, replication-competent virus testing, immune response, and manufacturing yield [122,134]. Extracellular vesicle-inspired systems require control of source material, purification, cargo loading, identity, purity, and biological activity [89].

**Figure 4:**
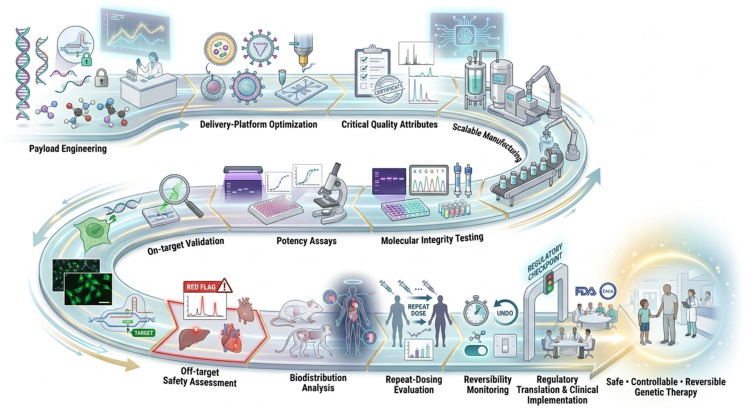
**Translational roadmap for reversible genetic-medicine delivery systems.** Roadmap illustrating the development pathway for reversible genetic-medicine delivery systems, beginning with payload engineering and delivery-platform optimization and progressing through critical quality attribute definition, scalable manufacturing, molecular integrity testing, potency assays, on-target validation, off-target safety assessment, biodistribution analysis, repeat-dosing evaluation, reversibility monitoring, regulatory translation, and clinical implementation. Created and edited using https://www.figurelabs.ai, accessed on 22 May 2026.

Regulatory translation requires clear documentation of product identity, manufacturing process, analytical methods, potency assays, safety testing, biodistribution, reversibility, and long-term monitoring [97]. Batch reproducibility must be demonstrated for both formulation attributes and functional editing performance. For a reversible claim, the development package should define the expected interval from dosing to payload clearance, the time course of molecular and phenotypic washout, the threshold for clinically meaningful recovery, and the conditions under which interruption, redosing, rescue, or counter-editing will be used. These endpoints should be supported by validated assays with prespecified acceptance criteria rather than by qualitative statements that the platform is transient. Regulatory strategy should also address delayed off-target effects, immunogenicity after repeat exposure, persistence in non-target tissues, and the consequences of incomplete reversal. The integrated development pathway from payload engineering and manufacturing control to potency assessment, safety evaluation, reversibility monitoring, and clinical translation is summarized in Figure 4.

## 9. Future Perspectives and Conclusion: Programmable Reversibility in Genetic-Medicine Delivery

Future development should convert reversibility from a descriptive label into an engineered and measurable product attribute. Payload-level temporal control can be achieved with short-lived mRNA or RNP formats, degradable carriers, inducible dimerization, destabilizing domains, regulated nuclear localization, guide-RNA decay, or orthogonal control circuits. RNA-guided control of DNA and chromatin provides a broader mechanistic foundation for programmable epigenetic regulation [135]. FIRE-Cas9, chemical-inducible Cas9, and inducible Cas13 systems illustrate how externally controlled assembly or activity can narrow the editing window [93,94,96]. CRISPRoff/CRISPRon and transient RENDER delivery further show that a temporary exposure can be programmed either to establish or to reverse epigenetic memory [23,24]. The next technical step is to integrate these controls with clinically usable triggers, low basal leakiness, rapid shutdown, and predefined rescue thresholds. 

A second priority is tissue- and cell-selective delivery beyond the liver. Clinically validated LNP delivery of Cas9 mRNA and guide RNA has produced substantial hepatic target reduction in patients [136], but the permanent genomic outcome in that study underscores the distinction between transient payload exposure and reversible therapeutic action. Extrahepatic programs will require route-specific formulations, ligand or receptor targeting, single-cell biodistribution and editing measurements, and evidence that non-target cells do not accumulate progressive molecular effects. AAV-mediated dCas9 repression can provide durable in vivo activity [137], yet vector persistence and immune barriers to redosing illustrate why high potency alone does not establish clinical controllability.

A third priority is prospective validation of reversal and rescue. Development programs should prespecify how an undesired effect will be attenuated: treatment withdrawal, guide blockade, pharmacological inactivation, opposing RNA or epigenome editing, transient expression of a counter-regulator, or replacement of the affected transcript or protein. Time-resolved multi-omic profiling should distinguish payload clearance from recovery of RNA sequence, chromatin state, transcription, protein abundance, and phenotype. These measurements should be linked to clinically interpretable kinetic endpoints, including time to peak effect, functional half-life, time to recovery, redosing interval, and cumulative immune or off-target burden.

In conclusion, reversible genetic medicines will be clinically credible only when delivery, duration, reversal, and manufacturing control are developed as one integrated system. Platform selection should be based on functional delivery to the relevant cell and compartment, a disease-appropriate exposure window, explicit separation of mechanistic, functional, and clinical reversibility, and validated options for interruption or rescue. Quantitative benchmarks remain heterogeneous and should be interpreted as study-specific rather than directly comparable across modalities. Rigorous kinetic assays, extrahepatic targeting, repeat-dose safety, and reproducible critical quality attributes will determine whether RNA-editing and epigenome-editing therapeutics can achieve programmable benefit without irreversible risk.

## Figures and Tables

**Table 1:** Reversible genetic-medicine payloads and delivery requirements.

Payload Class	Main Therapeutic Function	Key Delivery Requirement	Main Advantage	Main Limitation
ADAR-recruiting oligonucleotides	Site-directed RNA base editing using endogenous RNA-editing machinery	Nuclease protection, cellular uptake, endosomal escape, target-transcript access	Compact payload and potentially repeatable dosing	Editing depends on endogenous ADAR activity and transcript accessibility
Guide RNAs for RNA editors	Direct programmable recognition of target RNA	RNA stability, correct intracellular localization, co-delivery with editor machinery	High sequence programmability	Off-target RNA binding and degradation risk
CRISPR-Cas13-based RNA editors	RNA knockdown, RNA editing, or transcript modulation	Delivery of Cas13 machinery plus guide RNA, cytosolic or nuclear access	Programmable transcript-level intervention	Larger payload and potential immune activation
Chemically modified RNAs	Improved stability and reduced nuclease degradation for RNA-based modulation	Optimized modification chemistry, uptake, and intracellular activity	Enhanced stability and pharmacokinetic behavior	Excessive modification may reduce function or alter specificity
Editor mRNA	Transient intracellular production of RNA or epigenome editor proteins	Protection from degradation, endosomal escape, efficient translation	Transient expression and non-integrating format	Innate immune activation and limited tissue tropism
Self-amplifying RNA	Prolonged editor or regulatory-protein expression from lower input dose	Delivery of larger RNA, immune modulation, expression control	Longer expression at lower dose	Larger payload and higher immunostimulatory risk
Editor protein	Direct delivery of active editing enzyme or regulatory protein	Protein stabilization, cytosolic or nuclear entry, protection from degradation	Shorter activity window and improved temporal control	Poor intracellular delivery efficiency
Ribonucleoprotein complexes	Direct delivery of editor protein with guide RNA	Complex stability, cytosolic access, nuclear localization when needed	Rapid onset and reduced long-term expression	Large, fragile, and delivery-challenging payload
dCas9-based repressors	Targeted transcriptional silencing without DNA cutting	Nuclear delivery, guide RNA delivery, controlled residence time	Programmable gene repression	Large payload and possible off-target transcriptional effects
dCas9-based activators	Targeted activation of protective or deficient gene expression	Nuclear delivery and efficient chromatin targeting	Enables gene upregulation without DNA sequence change	Large multi-domain constructs may exceed delivery capacity
DNA methylation editors	Targeted epigenetic repression through methylation marks	Nuclear delivery and controlled persistence	Potentially durable gene silencing	Reversibility and off-target methylation require careful validation
Histone-modifying editors	Regulation of chromatin accessibility and transcriptional state	Nuclear delivery, chromatin access, temporal control	Fine-tuning of gene expression	Complex epigenome-wide safety assessment
CRISPRoff-like systems	Programmable epigenetic silencing with potential memory	Delivery of large editor constructs and guide RNAs	Durable repression without DNA cutting	Persistence may complicate reversibility
Plasmid DNA systems	Expression of RNA or epigenome editors	Nuclear entry, transcriptional activity, expression-duration control	Relatively stable and scalable payload	Prolonged expression may reduce reversibility
Viral vectors	Efficient tissue delivery of editor components	Packaging compatibility, tissue tropism, immune management	Strong delivery efficiency	Limited cargo capacity, immunogenicity, and repeat-dosing barriers
Lipid nanoparticles	Non-viral delivery of RNA, mRNA, or guide systems	Endosomal escape, tissue targeting, RNA stability	Clinically advanced RNA-delivery platform	Liver-biased distribution and inflammatory response risk
Polymeric or peptide carriers	Delivery of oligonucleotides, RNA, proteins, or complexes	Complexation, protection, uptake, intracellular release	Tunable chemistry and potential targeting	Toxicity and endosomal escape limitations
Extracellular vesicle-inspired systems	Biomimetic delivery of RNA or protein payloads	Loading control, targeting, reproducibility, purity	Biological compatibility and natural trafficking potential	Manufacturing and standardization challenges

**Table 2:** Payload-dependent functional delivery barriers and redosing constraints for reversible genetic medicines.

Payload-Carrier Context	Dominant Functional Barrier	Required Intracellular Access	Tropism or Route Consideration	Repeat-Dosing Practicality
ADAR-recruiting oligonucleotides or ASO-like guides	Nuclease protection, uptake, endosomal escape, and target-transcript accessibility	Usually cytoplasmic or nuclear transcript access depending on target RNA	Conjugation, local delivery, or non-viral nanoparticles; tissue uptake remains sequence- and chemistry-dependent	Generally more feasible than viral vectors, but limited by chemistry, renal clearance, and innate immune activation
Cas13 or editor-encoding mRNA with guide RNA	Co-delivery, RNA integrity, endosomal release, and controlled translation duration	Cytosolic access for translation and transcript targeting; nuclear access for nuclear RNA targets	LNPs are clinically advanced but often liver-biased; extrahepatic delivery requires formulation or route optimization	Possible with non-viral carriers if reactogenicity and anti-carrier responses are controlled
Protein or ribonucleoprotein editors	Large fragile complexes, serum stability, cytosolic entry, and rapid loss of activity	Cytosolic access for RNA targets; nuclear access for chromatin or nuclear RNA targets	Often suited to ex vivo, local, or strongly engineered nanoparticle delivery	Potentially transient, but repeated dosing must assess anti-editor immunity
dCas9-based epigenome editors	Cargo size, nuclear entry, chromatin access, and residence-time control	Nuclear and chromatin access are essential	Viral, mRNA, RNP, or local/ex vivo routes may be needed depending on cargo size	Limited when viral vectors are used; non-viral redosing remains technically challenging
AAV, adenoviral, or lentiviral vectors	Packaging capacity, expression persistence, and anti-vector immunity	Depends on encoded payload; nuclear expression is commonly required	Tropism can be strong but is capsid-, dose-, route-, and patient-immunity-dependent	AAV and adenoviral redosing is often constrained by pre-existing or induced immunity; integrating vectors are least compatible with reversibility
Hybrid or EV-inspired carriers	Loading efficiency, reproducibility, purity, and functional release	Payload-dependent; may support cytosolic or nuclear delivery if engineered appropriately	Potential for biomimetic or ligand-directed targeting, but standardization remains difficult	Potentially repeatable, but immune visibility and batch reproducibility must be demonstrated

**Table 3:** Disease applications, editable targets, and delivery strategies for reversible genetic medicines.

Disease Area	Representative Target or Mechanism	Suitable Reversible Approach	Delivery Strategy	Key Translational Challenge
Nonsense-mutation disorders	Premature stop codons in disease transcripts	RNA base editing or transcript repair	ADAR-recruiting oligonucleotides, RNA-editor mRNA, LNPs	Editing specificity and sufficient correction level
Splice-defect diseases	Aberrant exon inclusion/exclusion or cryptic splice sites	Splice modulation or RNA editing	Antisense oligonucleotides, chemically modified RNAs, nanoparticles	Tissue uptake and repeat dosing
Inherited metabolic disorders	Deficient hepatic enzymes or toxic metabolite pathways	Transcript correction, gene activation, or gene repression	LNPs, oligonucleotide systems, viral vectors	Durable but controllable hepatic expression
Liver diseases	Pathogenic hepatic transcripts, metabolic regulators, fibrosis genes	RNA editing or epigenetic repression/activation	LNPs, GalNAc-like targeting, polymeric carriers	Liver specificity and immune tolerability
Muscle diseases	Dystrophin-related transcripts, toxic repeat RNAs, splice defects	Splice correction, RNA editing, gene-expression modulation	Viral vectors, peptide carriers, LNPs, local injection	Broad muscle distribution
Inherited neurological disorders	Mutant transcripts, toxic RNAs, deficient protective genes	RNA editing, transcript knockdown, epigenome activation	Intrathecal delivery, viral vectors, engineered nanoparticles	CNS access and cell-type specificity
Cancer	Oncogenes, resistance genes, tumor-suppressive pathways	Transient oncogene repression, epigenetic reprogramming	Tumor-targeted nanoparticles, local depots, viral/non-viral systems	Tumor heterogeneity and off-target effects
Tumor microenvironment	Immunosuppressive cytokines, stromal genes, checkpoint pathways	Gene repression or immune modulation	Local nanoparticles, immune-cell-targeted carriers	Cell-specific delivery within complex tumors
Inflammatory diseases	Cytokines, inflammatory transcription factors, immune mediators	Transient gene silencing or epigenetic repression	LNPs, polymeric nanoparticles, local depots	Avoiding excessive immunosuppression
Autoimmune diseases	Autoreactive immune-cell programs, cytokine networks	Immune-cell reprogramming or cytokine modulation	Immune-cell-targeted nanoparticles, ex vivo modified cells	Maintaining immune balance and safety
Checkpoint regulation	PD-1, PD-L1, CTLA-4-related pathways, immune activation states	Reversible transcriptional control	Nanoparticles, viral vectors, cell-targeted systems	Avoiding systemic immune toxicity
Neurodegenerative diseases	Tau, alpha-synuclein, mutant huntingtin, neuroinflammatory genes	Transcript reduction, RNA editing, epigenome modulation	Intrathecal nanoparticles, viral vectors, EV-inspired systems	Blood–brain barrier and long-term safety
Cardiac remodeling	Hypertrophy, fibrosis, calcium-handling, inflammatory genes	Transient repression or activation	Cardiac-targeted nanoparticles, viral vectors, injectable depots	Cardiac tropism and dose control
Fibrotic diseases	TGF-β-related pathways, collagen regulators, myofibroblast genes	Reversible antifibrotic gene modulation	Local depots, tissue-targeted nanoparticles, LNPs	Avoiding impaired repair or off-target ECM effects
Age-related disorders	Senescence-associated secretory phenotype, mitochondrial pathways, inflammatory genes	Transient senescence or inflammation modulation	Nanoparticles, tissue-targeted oligonucleotides, local delivery	Context-dependent effects of aging pathways
Immune-cell therapies	T-cell exhaustion, macrophage polarization, antigen-presentation pathways	Ex vivo or in vivo immune-cell reprogramming	Viral vectors, electroporation, LNPs, polymeric carriers	Manufacturing complexity and immune safety

**Table 4:** Recommended potency, durability, reversibility, and safety endpoints by payload class.

Payload Class	Functional Potency Endpoint	Durability/Reversibility Endpoint	Minimum Safety Endpoint	CMC or Release Emphasis
ADAR-recruiting guides	On-target A-to-I editing and protein or phenotype rescue	Edited-transcript half-life and return toward baseline after guide clearance	Bystander editing in target RNA and transcriptome-wide A-to-I profiling	Guide purity, chemical-modification consistency, nuclease stability, and uptake
Cas13-based RNA editors	Target RNA knockdown, editing, or transcript modulation with functional rescue	Editor and guide persistence; recovery of target RNA after withdrawal when intended	Guide-dependent off-target RNA effects, altered splicing or RNA stability, and collateral activity where applicable	mRNA/guide integrity, co-delivery ratio, translation duration, and endosomal escape
Editor mRNA or self-amplifying RNA	Editor expression sufficient to produce intended RNA or epigenome modulation	Translation duration, dose–response, and decay of editor activity	Innate immune activation, cytokine induction, and off-target editing at clinically relevant doses	RNA integrity, capping, poly(A) quality, encapsulation, and storage stability
Protein or RNP editors	Rapid functional editing after direct delivery	Short editor residence time and loss of activity after clearance	Anti-editor immunity, cellular stress, and off-target editing from high intracellular exposure	Complex integrity, guide loading, activity, aggregation, and delivery efficiency
dCas9 epigenome editors	Target gene repression or activation with expected chromatin-state change	Persistence and reversibility of chromatin marks and gene-expression output	Genome-wide binding, DNA methylation or histone-mark spread, and transcriptome-wide perturbation	Cargo integrity, nuclear localization, guide specificity, and chromatin-target engagement
Viral-vector systems	Tissue transduction and editor expression sufficient for the intended effect	Expression persistence relative to the intended editing window	Anti-vector immunity, biodistribution, insertional risk when relevant, and long-term monitoring	Vector genome integrity, empty/full capsid ratio, infectivity, potency, and replication-competent virus testing
Non-viral nanoparticles and EV-inspired carriers	Functional intracellular release and payload-specific editing output	Carrier and payload clearance; repeat-dose reproducibility	Complement activation, cytokines, off-target biodistribution, and cell-type specificity	Particle size, polydispersity, loading, sterility, endotoxin, purity, and batch reproducibility

## Data Availability

No new data were created or analyzed in this study. Data sharing is not applicable to this article.
